# Vehicles of intercellular communication: exosomes and HIV-1

**DOI:** 10.1099/jgv.0.001193

**Published:** 2019-01-31

**Authors:** Jennifer L. Welch, Jack T. Stapleton, Chioma M. Okeoma

**Affiliations:** ^1^​ Department of Microbiology and Immunology, Carver College of Medicine, University of Iowa, Iowa City, IA 52242-1109, USA; ^2^​ Department of Internal Medicine, Carver College of Medicine, University of Iowa, 200 Hawkins Drive, Iowa City, IA 52242-1109, USA; ^3^​ Medical Service, Iowa City Veterans Affairs Medical Center, University of Iowa, 604 Highway 6, Iowa City, IA 52246-2208, USA; ^4^​ Department of Pharmacologic Sciences, Basic Sciences Tower, Rm 8-142, Stony Brook, University School of Medicine, Stony Brook, NY 11794-8651, USA

**Keywords:** HIV, semen, exosome, vesicle, virus

## Abstract

The terms extracellular vesicles, microvesicles, oncosomes, or exosomes are often used interchangeably as descriptors of particles that are released from cells and comprise a lipid membrane that encapsulates nucleic acids and proteins. Although these entities are defined based on a specific size range and/or mechanism of release, the terminology is often ambiguous. Nevertheless, these vesicles are increasingly recognized as important modulators of intercellular communication. The generic characterization of extracellular vesicles could also be used as a descriptor of enveloped viruses, highlighting the fact that extracellular vesicles and enveloped viruses are similar in both composition and function. Their high degree of similarity makes differentiating between vesicles and enveloped viruses in biological specimens particularly difficult. Because viral particles and extracellular vesicles are produced simultaneously in infected cells, it is necessary to separate these populations to understand their independent functions. We summarize current understanding of the similarities and differences of extracellular vesicles, which henceforth we will refer to as exosomes, and the enveloped retrovirus, HIV-1. Here, we focus on the presence of these particles in semen, as these are of particular importance during HIV-1 sexual transmission. While there is overlap in the terminology and physical qualities between HIV-1 virions and exosomes, these two types of intercellular vehicles may differ depending on the bio-fluid source. Recent data have demonstrated that exosomes from human semen serve as regulators of HIV-1 infection that may contribute to the remarkably low risk of infection per sexual exposure.

## Introduction

Cells release nanometre-sized lipid membrane particles that enclose various cell-associated nucleic acid and protein cargoes [1, 2]. The importance of cell-secreted vesicles in intercellular communication is clearly accepted. However, the terminology and distinguishing aspects for the wide variety of vesicles released from cells are much less clear. Such vesicles are commonly referred to as exosomes, extracellular vesicles, oncosomes, microvesicles and so on. Here, we will refer to them as exosomes for simplicity.

The wide range of definitions for cell-derived vesicle subtypes results in considerable overlap with the defining features of enveloped viruses. Functionally, viruses and cell-associated vesicles mediate intercellular communication by circulating, binding to and entering cells, and delivering their cargo to target or recipient cells [3]. In addition to similarities in function, exosomes and viruses are also similar in composition, potentially due to their overlapping use of biogenesis pathways. In infected cells, viruses and exosomes are produced simultaneously, resulting in the incorporation of viral material into exosomes. These features make differentiation and separation between the types of particles difficult. Distinguishing between exosomes and viruses in biological samples is important to understand their independent and dependent contributions to disease and identify potential therapeutic interventions. It is clear that exosomes may contribute to disease protection or pathogenesis, the reason for which is often unknown or cell-condition specific. Purification techniques aim to address this issue, however, all current methods of separation contain caveats that may influence downstream applications [4]. As a consequence of the range of defining characteristics, the lack of a universal marker, and the ability of exosomes to contain viral material, it is often impossible to validate homogenous separation from a heterogenous population.

In this review, we detail the current understanding of the similarities and differences of exosomes in general and enveloped viruses, emphasizing HIV-1, an enveloped retrovirus. Despite similar physical qualities, HIV-1 and exosomes may vary considerably in function, which may be attributed to exosome bio-fluid sources. Here we summarize the function of bio-fluid exosomes during HIV-1 infection. We focus on the association of exosomes and HIV-1 with semen, which is important during the sexual transmission of HIV-1. We emphasize the current understanding of semen exosomes and their role during HIV-1 infection. Because exosomes from semen regulate HIV-1 infection, we suggest that this subtype of vesicle may contribute to the low risk of HIV-1 sexual transmission per exposure.

### HIV-1/AIDS

More than 35 million deaths have been attributed to acquired immune deficiency syndrome (AIDS) since the disease was recognized in 1981 [5]. HIV-1, the causative agent of AIDS, is classified as a member of the genus *Lentivirus* in the family *Retroviridae* [6]. Although primate lentiviruses were known to exist at the time AIDS was identified, it is believed that the non-human primate form [simian immunodeficiency virus (SIV)] of HIV-1 was relatively contained within the simian population until a transmission event led to human infection through cutaneous or mucosa exposure [6, 7]. Subsequent viral adaptation to humans led to the emergence of HIV-1 and the resulting AIDS pandemic [7]. The isolation of HIV-1 from human semen and the ability of asymptomatic carriers to transmit the virus via cell-free and cell-associated seminal fluid contributed to the emergence of HIV-1 [8].

### HIV-1 replication

HIV-1 virions are ~100 nm spherical particles that contain an envelope (Env) comprising a lipid bilayer with intermittent viral glycoproteins [6]. These glycoproteins mediate HIV-1 cellular tropism and account for primary targeting of CD4+ cells [9]. The HIV-1 capsid contains two single-stranded copies of the RNA genome encased in the nucleocapsid, along with several viral enzymes and proteins [10]. The HIV-1 lifecycle begins with a binding/attachment event of the viral Env to the CD4 receptor on the host cell surface. HIV-1 further requires a co-receptor (CCR5 or CXCR4) for entry [9]. Binding to the viral receptor/co-receptor stimulates cell membrane fusion with viral Env, resulting in capsid entry, following which viral RNA is released into the cytoplasm. The HIV-1 single-stranded viral RNA is converted into double-stranded DNA (dsDNA) by the viral RNA-dependent DNA polymerase (reverse transcriptase). The resultant dsDNA is transported to the nucleus and integrated into the host cellular (chromosomal) DNA, equating to viral insertion into the host genome (proviral DNA). In activated, proliferating cells, proviral DNA replicates its genome utilizing host machinery for the transcription of viral RNA and translation of viral proteins. Accumulation of viral RNA and proteins leads to the assembly of new HIV-1 particles that are moved to the cell membrane. These new virions bud from the cell to result in new infectious particles [11]. A hallmark of HIV-1 pathogenesis is viral-mediated regulation of the host immune response and molecular pathways during infection [12]. As such, HIV-1 virions act as important vehicles in regulating intercellular communication.

### Non-viral vesicles released by cells

Cell-derived vesicles are important vehicles of intercellular communication that play significant roles in several pathologies, including cancer, neurodegenerative disorders and infectious diseases, such as viral infections. There are different types of cell-derived vesicles; however, for simplicity, we will refer to all cell-derived non-viral particles as exosomes throughout this review. Nevertheless, it is important to appreciate that cells produce diverse vesicles, often resulting in heterogenous vesicle composition. Cell-associated vesicles are characterized based on their origin, mechanism of release, size and potential markers (Table 1). The term extracellular vesicle is often used to generically describe most membranous cell-associated vesicles. However, by definition, extracellular vesicles (EVs) are a mixed population of exosomes, microvesicles, large oncosomes and apoptotic bodies [13, 14]. Exosomes account for small membranous vesicles that range in size from 40 to 100 nm, although this definition may evolve, as smaller non-membranous vesicles (~35 nm) have recently been described as exomeres [1, 15]. Exosomes exist as intraluminal vesicles within multi-vesicular bodies (MVBs) prior to release via the fusion of MVBs with the plasma membrane [1, 16]. Microvesicles are slightly larger, ranging from 100 to 1000 nm, while apoptotic bodies often account for 500–4000 nm vesicles. Both microvesicles and apoptotic bodies bud directly from the plasma membrane [17–19]. On opposite ends of the spectrum, large oncosomes range from 1 to 10 µm, whereas newly described exomeres exist as <50 nm (average 35 nm) vesicles. Large oncosomes and exomeres are distinguishable by their cancer cell association and non-membranous structure, respectively [14, 15]. These vesicles can also be classified based on originating cell type; for example, prostasomes specifically originate from the prostate epithelium and ectosomes often originate from monocytes and neutrophils [20, 21]. Although potential markers and cargoes are described for each vesicle classification, a large overlap between nucleic acid and protein content generally exists. This overlap in multiple characteristics of cell-associated vesicles has resulted in the frequent use of nonspecific terminologies.

**Table 1.** Classification of cell-associated vesicles

Classification	Origin	Mechanism of release	Size	Potential markers	Source*
Extracellular vesicles	Mixed population of exosomes, microvesicles, apoptotic bodies, large oncosomes	Fusion of MVBs and direct budding from the plasma membrane	Variable 40 nm–10 µm	Varied: tetraspanins, major histocompatibility complex (MHC) molecules, cytosolic proteins	[13]
Intraluminal vesicles	Multiple cell types (endosome-associated)	Intraluminal vesicles exist within MVBs and upon release these vesicles are termed exosomes	40–100 nm	MHC II, tetraspanins, ubiquitinated proteins	[1] [16]
Ectosomes	Multiple cell types (commonly neutrophils or monocytes)	Plasma membrane budding	100–350 nm	TyA, C1q	[21]
Exosomes	Multiple cell types (endosome-associated)	Fusion of MVBs with plasma membrane	40–100 nm	CD9, CD63, CD81, TSG101, Alix, Hsp70	[1] [15]
Exomeres	Multiple cell types	Not yet described	<50 nm	Non-membranous, Hsp90-β	[5]
Prostasomes	Prostate epithelium	Budding from the plasma membrane of prostate epithelial cells	40–130 nm	PAP, PSA, TMPRSS2, PSCA	[20]
Microvesicles	Multiple cell types	Plasma membrane budding	100–1000 nm	Phosphatidylserine, integrins, selectin, CD40	[17] [18]
Oncosomes/ large oncosomes	Tumour cells	Cancer cell membrane budding	1–10 µm	EGFRvIII, ARF6, Cav-1, CK18, oncogenic material	[107] [14] [23] [14]
Apoptotic bodies	Cells undergoing apoptosis	Blebbing and fragmentation of the plasma membrane of apoptotic cells	500–4000 nm	Phosphatidylserine, annexin V, thrombospondin, C3b	[19]
Enveloped virus particles	Virally infected cells	Plasma membrane budding	~100 nm	Viral-encoded proteins	[22]

*The cited references are not an exhaustive list. We apologize to authors whose work was unintentionally omitted.

### Exosomes and enveloped viruses

By strict definition exosomes are different from viruses because of their inability to replicate their contents. Unlike viruses, exosomes are metabolically inert and cannot reproduce their contents to generate progeny from producer cells [22]. However, as previously reviewed, exosomes and viruses do not conform to strict definitions [22]. Intermediate particles exist on the spectrum between virus and exosome that contain both host and viral components, making it nearly impossible to classify these vesicles as either defective viruses or exosomes that contain viral components [22]. Intermediate particles are often classified as a virus or exosome derivative, depending on the preference of the investigator, but once these vesicles deviate from strict definitions they may be more accurately defined as an assortment of lipid-encased particles that cannot be easily differentiated [22].

There are structural and functional similarities between exosomes and enveloped viral particles. Although heterogeneous, exosomes are similar in size to retroviruses (~120 nm), and the biogenesis of both exosomes and viral particles involve shared cellular pathways [22]. An important difference between exosomes and enveloped viral particles is the ability of enveloped viral particles to replicate their contents. Although exosomes may contain virus-associated nucleic acids and proteins, true exosomes do not replicate [22]. However, the recent discovery of quasi-enveloped hepatitis E and A viral particles complicates this distinction. In the case of these non-enveloped viruses, exosomes provide a pseudo-envelope that allows the particles to transmit infection [23–25]. Similarly, exosomes carrying infectious hepatitis C virus RNA can transmit infection [26]. However, in these cases it may be argued that these virus RNA-containing exosomes are more similar to viral particles that have hijacked exosome biogenesis pathways than typical, cell-derived exosomes. These intermediate exosome/viral particles have been reviewed elsewhere [22, 27]. Due to the inherent nature of exosomes to reflect the status of the producer cell at the time of biogenesis, the role of exosomes during HIV-1 infection is diverse [28]. Simply put, exosomes contribute to viral pathogenesis by regulating cellular functions that may inhibit or enhance viral infection [28]. The secretion of exosomes from nearly all cell types and their detection in a variety of biological fluids illustrates their wide-reaching influence on the cell microenvironment. Further, the localization and cellular derivation contributes to the complexity of identifying specific exosome subtypes and the exosome-conveyed properties responsible for modulating host functions [29].

### Factors influencing HIV-1 sexual transmission

Vaginal and rectal sexual intercourse accounts for >70 % of HIV-1 infections worldwide [30]. During mucosal exposure, HIV-1 infection often occurs via transfer of virus from dendritic cells (DCs) to monocytes/macrophages, while direct infection of CD4+ T cells is more likely during parenteral transmission [6]. It is important to note that during mucosal infections, HIV-1 must traverse the layers of the epithelium to reach these target cells [31]. However, during HIV-1 sexual transmission, intercourse may result in trauma to the epithelial cell layer, leading to increased access to CD4+ cells [31]. Although sexual transmission accounts for the majority of infections worldwide, the risk of contracting HIV-1 per exposure is far greater during blood exposure than with semen exposure (risk/exposure: 9250/10 000 from blood transfusion, 63/10 000 from needle sharing and 8/10 000 vaginal intercourse) [32]. While blood components may influence infection, semen is the major transmission vehicle during sexual transmission [31]. The high viral loads contained in the semen of HIV-infected individuals (up to 1.3×10^7^ RNA copies ml^−1^) suggest that semen may transmit HIV-1 via both seminal CD4+ cells or as cell-free virus [33, 34]. Despite semen serving as a carrier of HIV-1, individual seminal components have different roles during HIV-1 transmission and may facilitate or inhibit infection [35, 36]. Semen is highly complex, with more than 900 proteins and additional carbohydrates and lipids [35, 36]. This may explain the finding of both pro- and anti-HIV-1 factors in semen.

### pH regulatory and immunomodulatory role of semen

The immunomodulatory effects of semen alter inflammatory signalling pathways and mucosal barrier integrity, and are crucial for reproductive success [37]. In addition to aiding reproduction, these functions can alter susceptibility to HIV-1 infection during sexual transmission. For example, the normal vaginal pH is acidic (4.0 to 6.0), reduces sperm viability and motility, and is hostile to HIV-1 particles that are inactivated at pH<5.0 [31, 38]. However, semen is alkaline and raises vaginal pH to the range of 6.0 to 7.0 [31], potentially favouring fertilization and HIV-1 infection [38, 39]. Aside from pH changes, some factors contained in semen promote disruption of the mucosal barrier, thereby enhancing HIV-1 infection of immune cells that reside in the subepithelial layer of the reproductive tract. Typically, HIV-1 virions become trapped in the mucosal barrier and are less efficient at diffusing through intact epithelial layer [40].

### HIV-1 enhancing immune factors of semen

In addition to supporting spermatozoa by promoting liquefaction of the coagulated fluid increasing spermatozoa motility, semen is also associated with modulation of immune responses within the reproductive microenvironment [41]. Semen, in comparison to blood, contains increased levels of inflammatory cytokines regardless of HIV infection status, although specific cytokines appear to be up-regulated during HIV-1 infection [35]. Semen induces cellular secretion of the inflammatory cytokines/chemokines IL-8, MCP-1, IL-6, GM-CSF, CCL20 and others. These factors may function in HIV-1 target cell recruitment, maturation and activation [31, 36]. The enrichment of TGF-β, PGE2 and IL-7 in semen is thought to induce both pro- and anti-inflammatory responses in a variety of immune cells. This may influence cell influx and act to suppress host immunity, thus facilitating HIV-1 replication [31, 42]. It is postulated that seminal cytokines may influence inflammatory changes in order to support tolerance in pregnancy, as the introduction of material into the female reproductive tract would typically elicit an immunogenic response [43]. Perhaps the pro-survival properties of semen that support spermatozoa within the female reproductive tract also promote the survival of HIV-1 virions during infection [44]. Semen CCL2 levels negatively correlate with CD4+ T cell counts, and seminal HIV-1 RNA concentration correlates positively with semen IL-6, IL-16, CCL2, CCL11 and CXCL12b [44]. This finding has been reproduced in a macaque model of SIV transmission [45], suggesting that seminal-associated cytokines promote HIV-1 infection.

### HIV-1 inhibitory factors in semen

The cytokine/chemokine profile of semen may confer antiviral activity during infection. It has been suggested that CCR5-binding cytokines and CXCR4 ligands in semen may suppress the replication of CCR5- and CXCR4-tropic strains of HIV-1, respectively [46]. Moreover, an unidentified component of semen inhibits HIV-1 infection of DCs by inhibiting viral attachment to DC-SIGN, an important mode of HIV-1 entry [36, 47, 48]. Semen clusterin was recently implicated in this inhibition, as it binds to DC-SIGN and competes for HIV-1 binding [49]. Clusterin is a glycoprotein that is expressed in many tissues in either a secreted or a nuclear-associated form, and it is implicated in numerous functions [49]. Semen clusterin contains an abundance of fucosylated N-glycans with a high binding affinity for DC-SIGN, as shown by the inability of deglycosylated and defucosylated semen clusterin to inhibit the binding of HIV-1 to DC-SIGN [49]. Although semen clusterin is potent against HIV-1, the depletion of clusterin did not completely restore HIV-1 binding to DC-SIGN [49], suggesting the presence of multiple DC-SIGN ligands within semen that may contribute to the inhibition of HIV-1 attachment. Indeed, it has been shown that seminal mucin-6 inhibits HIV-1 attachment to DCs and subsequent DC-mediated transfer of HIV-1 to CD4+ T cells [50]. Further, semen has been shown to inhibit HIV-1 infection of CD4+ T cells [51].

### Pro- and anti-HIV factors in semen

Whether semen functions to promote or inhibit infection depends on multiple factors, including the immunomodulatory factor content, the immune or inflammatory status of the reproductive tract, HIV-1 viral load and antiretroviral therapy (ART) [35]. It is currently unknown how exactly the factors present in semen regulate HIV-1 transmission. Semen factors may stimulate target cell recruitment, activation and/or break-down of the mucosal barrier, thereby increasing infection, or reduce susceptibility to infection by cytokine/chemokine expression or blocking viral attachment to DCs [35]. The early hypothesis that semen increased HIV-1 transmission centred on the presence of semen-derived enhancer of viral infection (SEVI) detected *in vitro* [52]. SEVI appears to be fragments of prostatic acidic phosphatase that, through the formation of amyloid fibrils, capture HIV-1 virions and increase viral transmission *in vitro* [52]. A similar attribution is assigned to semenogelin fibrils (SEM1 and SEM2) [53]. However, the relatively low frequency of heterosexual HIV-1 transmission contradicts the idea that semen greatly enhances transmission, especially given that the risk of infection per act of coitus varies between 0.0001 and 0.001 [54].

**Fig. 1.**
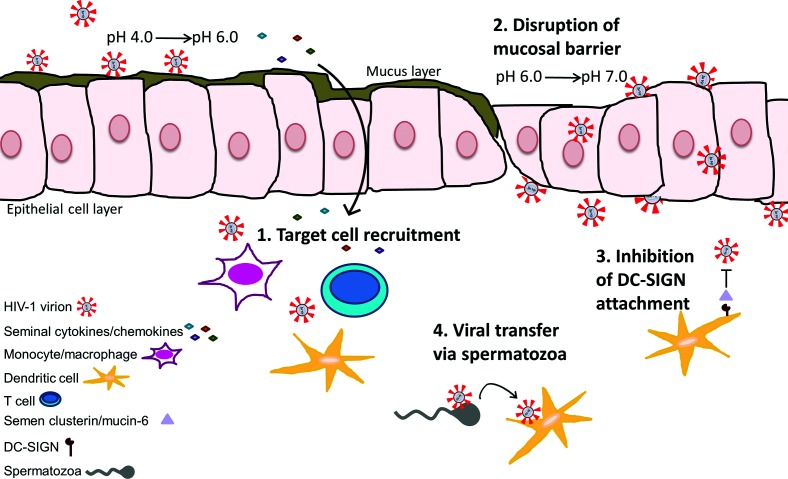
Schematic representation of the effects of semen on the mucosal microenvironment during HIV-1 transmission. (1) Seminal-associated cytokines/chemokines may traverse the mucosal layer and recruit cells susceptible to HIV-1 infection, such as CD4+ T cells, dendritic cells and monocytes or macrophages. (2) The alkaline properties of semen raise the acidic vaginal pH from a range of 4.0–6.0 to one of 6.0–7.0, disrupting the protective mucosal layer and allowing more efficient viral dissemination through the epithelial cell layer. (3) Clusterin and mucin-6 molecules in semen prevent HIV-1 attachment to DC-SIGN on dendritic cells. (4) Spermatozoa may act as carriers of HIV-1 virions to susceptible cells. See text for references.

The presence of two forms of infectious HIV-1 in semen, cell-free and cell-associated virus, contributes to its role in facilitating viral transmission, as both forms have advantages for overcoming mucosal barriers [40]. Predominately, cell-free virus passively diffuses through the extracellular space to reach distal cells, whereas cell-associated virus is more rapid and efficient at infecting susceptible cells [40, 55]. Spermatozoa enhances HIV-1 infection, especially of DCs, but also of macrophages and CD4+ T cells [31]. Spermatozoa appears to enhance the efficiency of virus attachment to target cells when compared to cell-free virus [31]. It is unclear whether HIV-1 is able to bind to and enter spermatozoa, and this has been a source of debate despite the identification of HIV-1 nucleic acids within spermatozoa obtained from HIV-1 infected men [56]. Mannose receptors, glycolipids and heparan sulfate on spermatozoa are proposed as potential receptors for HIV-1 binding due to their ability to interact with HIV-1 gp120 [56]. Similar to the viral capture function described for spermatozoa, the presence of soluble complement components within semen appear to opsonize HIV-1 and enhance infection of epithelial, monocyte/macrophage, T and B cells *in vitro* [57]. Additionally, semen contains an inhibitor of the complement pathway *in vitro*, as CD59 aids escape from complement-mediated lysis [40, 58]. However, the role of complement during HIV-1 infection is controversial, making the contribution of semen to the complement system during infection unclear. *In vivo,* the role of semen during HIV mucosal infection is complex and likely a summation of multiple effects [59], as depicted in Fig. 1.

**Fig. 2.**
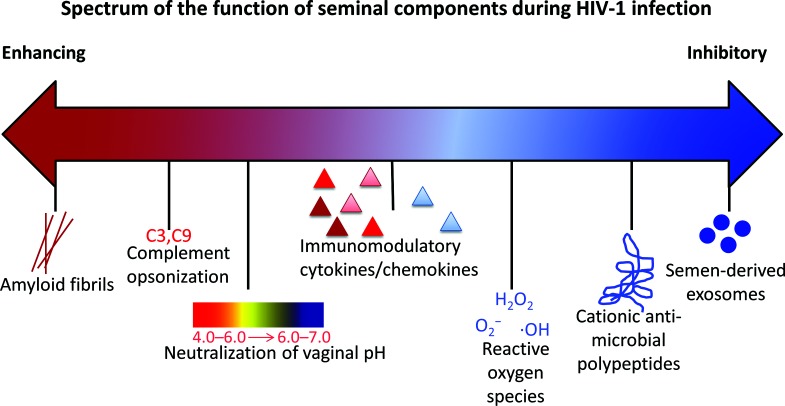
Spectrum of the function of seminal components during HIV-1 infection. HIV-enhancing components (red) and HIV-inhibitory components (blue) co-exist in seminal fluid. See text for references.

The presence of reactive oxygen species and cationic antimicrobial peptides are factors suggested to explain the HIV-inhibitory phenotype of semen, as these inhibit HIV-1 infection *in vitro* [36]. Nevertheless, the enhancing and inhibitory properties of amyloid fibrils, cationic peptides and reactive oxygen species during HIV-1 infection are controversial [60, 61]. More recently, the role of exosomes has been investigated to understand the function of blood and semen during HIV-1 transmission. Using a variety of cell models and a murine AIDS model, exosomes derived from human semen were shown to inhibit HIV-1 infection, raising the possibility that anti-viral exosomes within semen may contribute to the low frequency of sexual HIV-1 transmission [62–64]. To summarize, semen likely contains a spectrum of components with variable functions that influence HIV-1 transmission, but the relative semen components and their activity are not clearly understood (Fig. 2).

### Non-semen body fluid exosomes and HIV-1

In recent years, the exosomes field has exploded, with new exosome-attributed functions being described during the progression of cancer, the spread of pathogens, immune regulation and normal cell development and differentiation. For example, *in vivo*-derived serum exosomes transmitted infectious human pegivirus (HPgV; previously GB virus C/hepatitis G virus) RNA to primary blood mononuclear cells (PBMCs) *in vitro* [65]. Cell culture-derived exosomes from HPgV-infected cells delivered HPgV protein to natural killer (NK) cells that inhibited NK cell immune functions [66]. Similarly, human serum-derived exosomes containing hepatitis C virus (HCV) RNA transferred HCV RNA to PBMCs and interfered with T cell receptor (TCR) signalling [67]. Thus, the biological impact of exosomes is widespread and significant [68]. Initially, it was not clear if exosomes represented cell debris or an artifact of the experimental systems in which exosomes were detected. Subsequent studies demonstrated the enrichment of particular cell-associated proteins regardless of the isolation strategy or cell type, confirming that exosomes are the result of distinct cellular processes giving rise to a distinct population of cellular vesicles [69]. A ubiquitous exosome marker has yet to be formally assigned, but specific proteins such as CD9, CD63, CD81, HSP70, HSP90, MHC I and II, and acetylcholine-esterase are commonly found in exosomes, and thus may serve as useful exosome indicators [17, 64]. The composition, cargo and resulting function of exosomes rely on the status of the producer cell; therefore, the ever-changing condition of the cell dictates exosome composition and biogenesis, precluding the use of a specific protein to identify all types of exosomes.

### ESCRT-dependent and -independent pathways, HIV-1 and exosome biogenesis

Endosome compartments and the endosomal sorting complex required for transport (ESCRT) pathway are essential for exosome secretion [69]. Briefly, exosomes are formed by the endocytosis of plasma membrane proteins into early endosomes. Endosomes mature into MVBs, where invaginations into MVBs result in the formation of individual vesicles that acquire sorted proteins, lipids and nucleic acids [3]. Invaginations into MVBs result in the incorporation of cytosolic components into individual vesicles, particularly cytosolic RNA species (mRNA, miRNA and non-coding RNA) [70]. The incorporation of RNA cargo into vesicles is highly dependent on the physiological state of the cell; therefore, the RNA cargo profile of vesicles may differ from the profile of the originating cell [3]. It is suggested that particular 3′UTR mRNA sequences may be preferentially targeted into vesicles [71]. The lipid composition of vesicles often closely resemble the composition of the originating cell, although polyunsaturated glycerophosphoserines and phosphatidylserines seem to be particularly enriched in vesicles [3]. The vesicles’ lipid casing is derived from the lipid membrane of MVBs during the invagination step, at which time cytosolic lipids are also encased in vesicles [3, 70]. Vesicle escape into the extracellular domain from the producer cell occurs via MVB exocytosis [68]. Exosome biogenesis may occur through ESCRT-dependent and -independent mechanisms, but the ESCRT pathway is the most well understood mechanism [72]. Like exosome release, the ESCRT pathway is also required for HIV-1 budding. The HIV-1 Gag structural protein binds components of the ESCRT pathway to promote budding from the plasma membrane as the major route of viral egress [73]. For both exosomes and HIV-1 virions, utilization of the ESCRT machinery results in the accumulation of a lipid bilayer from budding or fusion events with the plasma membrane [73].

MVBs are able to form in cells depleted of ESCRT components. During ESCRT-independent exosome secretion, ceramide formation, tetraspanins, phospholipase D2 and ADP ribosylation factor-6 mediate vesicle formation [72, 74]. ESCRT-independent pathway events may contribute to viral spread and to immune modulation during viral infections. ESCRT-independent domains alter the sorting of proteins and nucleic acids into exosomes, including the packaging of viral components into exosomes. For example, the tetraspanin CD63 mediates the sorting of Epstein–Barr virus latent membrane protein 1 (LMP1) into intraluminal vesicles and packaging into exosomes [75]. The ESCRT-independent pathway may also facilitate the sorting of viral components into exosomes of viruses that rely on ESCRT components for release, possibly increasing the ability of these viruses to be released from cells. Exosome delivery of HSV-1 tegument proteins may ‘prime’ cells for infection by immediately activating transcription upon contact with infectious virions. In addition, the packaging of viral components such as HIV-1 Nef protein in exosomes enhances the ability of HIV to evade immune recognition by suppressing antiviral responses in recipient cells [76]. Exosomes and viruses exploit ESCRT-dependent and -independent pathways for biogenesis events.

### Exosomes and HIV-1 overlap in composition

Due to the utilization of the same cellular pathways, exosomes and newly synthesized HIV-1 virions incorporate similar molecules, including tetraspanins [77], multi-vesicular body-associated proteins [78] and cytoskeleton proteins [79, 80]. In addition, exosomes isolated from HIV-1-infected sources contain viral materials, including the viral trans-activation response element (TAR) RNA and proteins such as Nef and Gag. Transport of these viral factors to permissive cells facilitates infection *in trans* [81–84]. The distinction between exosomes and HIV-1 particles is made even more ambiguous by the existence of human endogenous retroviruses (HERVs). HERVs are evolutionarily ancient non-coding and protein coding retroviral sequences within the human genome that are unable to produce infectious virions. Specifically, HERV components, including HERV-associated reverse transcriptase (RT), RNA, Gag and Env proteins, are found in human exosomes [76]. HERV sequence-containing exosomes can facilitate the transfer of HERV mRNA to cells *in vitro* [76]. These are only a few of the many constituents shared by viral particles and exosomes, and they highlight the shared mechanisms of biogenesis between the two particle types.

Upon release from the producer or infected cells, exosomes and viral particles share features regarding how they interact with other cells via protein binding, endocytic pathway uptake and membrane fusion [9, 85]. Following cellular entry, exosomes and HIV-1 virions act as delivery vehicles for information. Both disperse their contents into cells and influence biological processes, frequently by appropriating cellular machinery [76]. HIV-1 virions could be described as exosomes that are unique in their ability to replicate their contents. However, this is controversial based on the definition of replication, such as in the case of replication-incompetent viruses. These particles are still considered to be HIV-1 virions, but by definition they are unable to reproduce their contents in living cells. Nevertheless, the similarities raise the question of whether or not HIV-1 virions are simply modified exosomes [22]. This question has led to the development of the Trojan exosome hypothesis [76], which reasons that exosomes and retroviruses contain extensive overlap in characteristics because retroviruses use the exosome pathway to facilitate receptor-independent infection [86]. Since the formation of retroviruses is driven by Gag protein expression, the interaction of Gag with intraluminal vesicles directs retroviruses to the exosome biogenesis pathway for the formation of infectious virions [86, 87]. While the Trojan exosome hypothesis warrants consideration in understanding HIV-1 strategies outside of the classical model of receptor/co-receptor cellular infection and may offer value for understanding phenotypic similarities between exosomes and HIV-1, others argue that using the same cellular pathways does not make HIV-1 virions modified exosomes [88].

### Function of body fluid exosomes during HIV-1 infection

It is important to again stress that exosomes may facilitate or inhibit HIV-1 infectivity, and that the effect is influenced by the producer cell of origin [28, 76]. Because biological fluid exosomes, such as those derived from blood or plasma, originate from multiple cell types, proviral or antiviral effects may be present in different fluids or from different donors [89]. For instance, blood- or cell culture-derived exosomes transfer the HIV-1 co-receptors, CCR5 [90] and CXCR4 [91], offering the ability to transform HIV-1-resistant cells into HIV-1-susceptible cells, depending on cell expression of CD4 receptor. In addition to HIV-1 nucleic acids and protein [84, 92, 93], exosomes can also transfer cellular or viral factors that down-regulate the immune response to infection or enhance inflammatory signals [94, 95]. Exosomes from diverse biological systems contain surface-associated and encapsulated biologically active cytokines [96]; blood exosomes from HIV-1 patients contain cytokines/chemokines that increase the activation levels of CD4+ and CD8+ T cells [97]. Exosomes from CD4+ T cells reactivate latent-SIV CD4+ T cells in a macaque model, which is potentially important for therapeutic applications targeting latent viral reservoirs [98]. Conversely, exosomes containing antiviral compounds such as APOBEC3G and interferon α/β, and exosomes from CD8^+^T cells suppress HIV-1 infection *in vitro* [99–102]. Exosomes derived from CD4^+^T cells that contain CD4 inhibit HIV-1 infection compared to CD4-depleted exosomes [103]. In addition, exosomes derived from human breast milk [104], vaginal fluid [105] and semen [62–64] exhibit potent anti-HIV activity. Urine, saliva and ascites fluid exosomes have yet to be explored in HIV-1 infection, although proteomic analysis of saliva from HIV-positive heroin addicts identified that HIV infection modified the cargo of exosomes [89, 106, 107]. Remarkably, a head-to-head comparison of blood-derived exosomes with those purified from either breast milk [104] or semen [63] showed opposing functions. Blood-derived exosomes had no effect or enhanced infection while breast milk- and semen-derived particles consistently inhibited infection [63, 104]. The proviral and antiviral features identified in exosomes highlights the fact that exosome composition may play an important role in cellular permissiveness and susceptibility during HIV-1 infection. However, caution should be used when comparing exosome studies, as a multitude of factors may influence observations, including the exosome isolation protocol [108, 109], sample storage conditions [64, 110] and the efficacy of recipient cell uptake [62, 111].

### Separating virus from exosomes

The overlapping features of exosomes and HIV-1 particles makes the purification of exosome and virus populations from the same source difficult if not impossible, complicating determination of the composition and functions of exosomes during different stages of HIV-1 infection. Popular techniques rely on velocity gradient separation such as iodixanol, since the density of HIV-1 virions and exosomes are somewhat different, although there is considerable overlap (1.13–1.21 g l^−1^ for exosomes and 1.16–1.18 g l^−1^ for HIV-1) [4]. Thus, these techniques can be unreliable due to the similarity in biophysical properties and heterogenous nature of exosomes. Immuno-depletion or immuno-capture techniques have been suggested as ways to purify and concentrate exosomes from HIV-1-containing sources. Here, anti-acetylcholinesterase- and/or anti-CD45-coated beads are used to capture exosomes without binding to HIV-1 [112]. Theoretically, this technique is a means to concentrate pure exosomes from HIV-1 particles without the addition of substances influencing down-stream HIV-1 functional assays. However, as at the time of this review, there is presently no way to remove exosomes bound to affinity beads without destroying exosome integrity, including exosome surface-associated molecules. Further, immuno-depletion or immuno-capture techniques may exclude some exosomes that are surface protein-negative (or double negative) and are still capable of affecting functional studies. Exosome subpopulations contain variations in surface composition that may affect function; for example, CD63 surface protein levels from human semen-derived exosomes correlated to the inhibition of HIV-1 infection, where semen exosomes with reduced surface CD63 showed diminished ability to inhibit HIV-1 infection [64]. Similarly, depletion of the CD63-positive exosome population in herpes simplex virus-1-infected cells enhanced infection [113]. Thus, efficient methods of exosome and HIV-1 separation that maintain virion and vesicle integrity without the complication of functional assays are needed.

### Therapeutic applications

Although there are difficulties in separating exosomes and retroviral particles (including human and murine retroviruses and retrotransposon elements), each particle type has the ability to transfer materials to cells. This has been exploited for use in therapeutic delivery systems. Both exosomes and retroviral vectors are being used to deliver immunotherapies and gene therapies because of their capacity to act as efficient transporters of bio-information [114, 115]. Both particle types provide a stable vehicle to encapsulate cargo with reduced immunogenicity [114], historically a major problem in similar delivery systems. Exosomes and lentiviruses are also proficient at interacting with multiple cell types and across tissue barriers, including the blood–brain barrier [116, 117]. The surface properties of both types of particles can be modified to allow for targeted interactions [116, 118]. Despite the similarities, exosomes and lentiviral vectors have important differences. The inherent capacity of exosomes to enclose nucleic acids, proteins and lipids allows them to package biological and chemical agents [116]. Consequently, exosomes are employed in drug delivery systems, which is not a practical feature of lentiviral vectors. Although exosomes have been considered for use in gene therapy, particularly to carry coding and non-coding RNA, including regulatory RNAs (miRNA and siRNA), lentiviral vectors have significant advantages in this therapeutic market because of their ability to confer stable integration into target cells [119, 120]. The ease of engineering and non-synthetic nature of these delivery systems offers advantages for disease targeting, and both approaches are currently being tested in human clinical trials (https://clinicaltrials.gov).

### Exosomes as biomarkers

Exosomes are a ‘fingerprint’ of the cell condition; thus, circulating exosomes are considered to be potential biomarkers of disease [121]. Exosomes are considered to be advantageous biomarkers due to their stability, sensitivity and specificity [122]. Cancer cell-derived exosome nucleic acid content may act as tumour markers. For example, the plasma- or urine-derived exosomes survivin, PCA-3 and TMPRSS2:ERG are associated with prostate cancer [123]. Additionally, the proteomic profile of seminal plasma reveals potential markers of male infertility, such as semenogelins, protein DJ-1, prostatic acid phosphatase, kallikrein 3 and prolactin-inducible protein, to name a few [124–127]. Exosomes as markers of cancer and non-cancer disorders have been reviewed elsewhere [121, 128, 129]. Although studies are limited, exosomes containing viral material may be considered to be a marker of viral infections. Exosome-associated immune and oxidative stress markers were evaluated as indicators of HIV-1 disease progression. In addition to an increased abundance, plasma exosomes of HIV-positive ART-suppressed patients showed increased oxidative stress markers, reduced anti-inflammatory polyunsaturated fatty acids (PUFA) and increased inflammatory response regulator, Notch4, compared to HIV-negative patients [130]. In a separate study, HIV-positive ART-naïve patients contained an increased abundance of plasma exosomes that were larger in size with enhanced miRNA levels than in HIV-positive ART-suppressed patients [131]. Exosome abundance and size correlated inversely with CD4-T cell counts and correlated positively with CD8-T cell counts [131]. These studies may indicate that exosomes serve as indicators of oxidative stress, immune activation and inflammation during HIV-1 progression.

### Semen exosomes and HIV-1

**Fig. 3.**
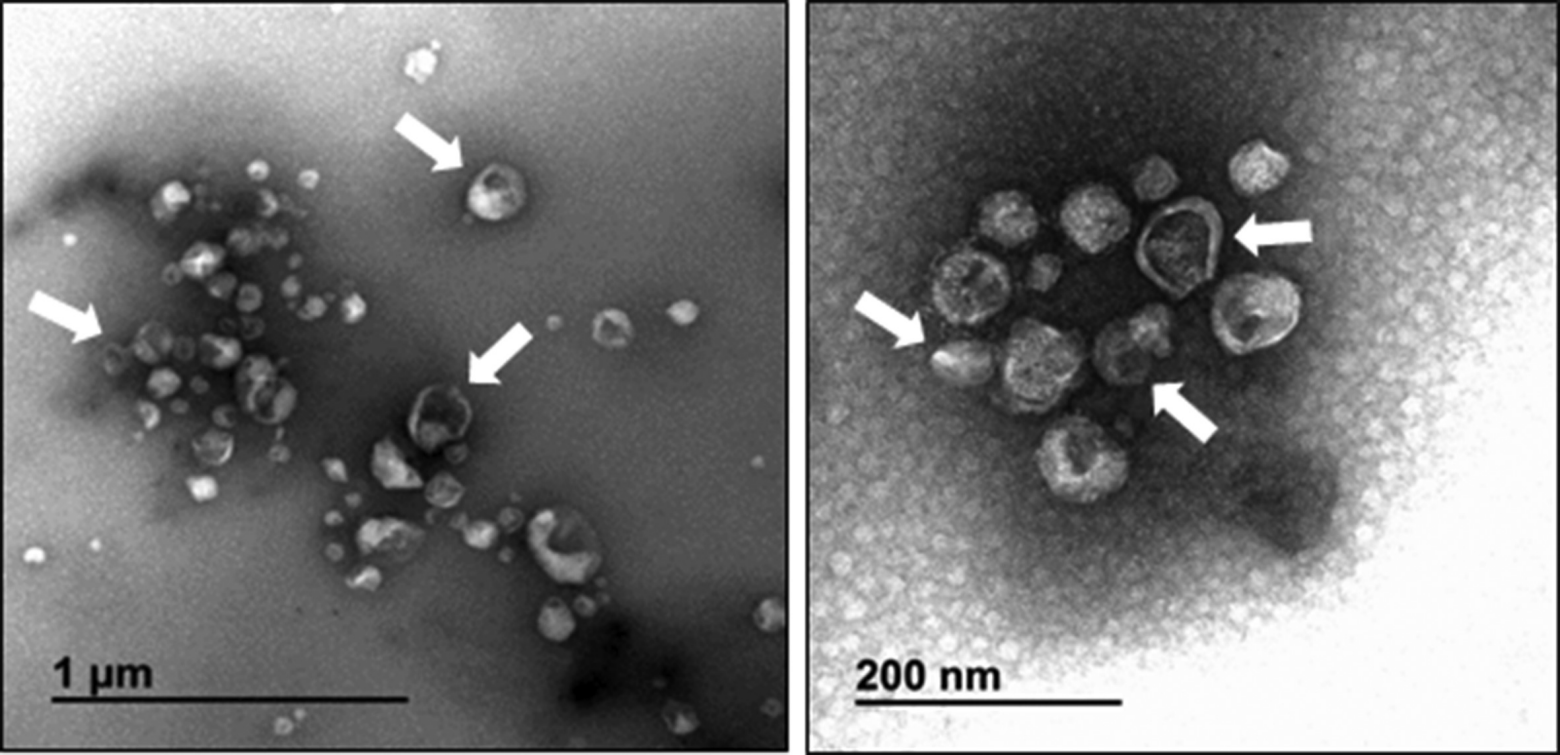
Electron micrograph of semen exosomes obtained by negative staining. Shown is a heterogeneous population of vesicles consisting of a range of sizes with singular or double membranes of differing densities (translucent light vs translucent dark particles). The white arrows highlight a few exosome particles.

During HIV-1 transmission, exosomes within human semen coexist with the virus in the male reproductive tract before widespread dissemination within the newly infected host; thus, biofluid-specific exosomes may provide novel insights into the role of exosomes in sexual transmission. Semen exosomes (SEs) are a heterogeneous population (~30–200 nm in diameter) of extracellular vesicles that likely includes exomeres, generalized as exosomes for simplicity. SEs are composed of a range of morphologies and electron densities (Fig. 3) [63, 64]. SE abundance is estimated to range from 10^11^ to 10^12^ particles ml^−1^semen. Since the average human ejaculate of semen is approximated at 3.7 ml, SEs are highly concentrated [64, 132, 133]. In comparison, the concentration of blood exosomes is approximately 10^9^–10^10^ vesicles ml^−1^ plasma [134, 135]. Characteristic of other exosomes populations, SEs contain cell surface-associated proteins shared by many exosomes, including CD63, CD81, CD9 and acetylcholine esterase, in addition to protein and nucleic acid cargoes. Cargoes may include small RNAs and mRNA capable of supporting or regulating gene expression [63, 64].

SEs are internalized into cells by endocytosis and fusion with the cell membrane; treatment of cells with an inhibitor of macropinocytosis did not affect SE internalization, indicating that macropinocytosis is not a major route of cell entry for SEs [62]. Comparison of the uptake efficiency of SEs and blood exosomes revealed that vaginal epithelial cells internalize SEs more efficiently than blood exosomes by an order of magnitude; however, blood exosomes are more efficiently internalized in monocytic (U937) and lymphocytic (SUPT1) cell lines than SEs [62, 63]. This internalization efficiency may contribute to SE interference with HIV-1 infection of epithelial cells within the female reproductive tract by reducing HIV-1 transcytosis across the mucosa to reach CD4+ target cells. In both a co-culture and a transwell model of HIV-1 infection, SEs blocked the transfer of HIV-1 from vaginal epithelial cells to monocytic (U937) and lymphocytic (SUPT1) cell lines, thus SEs appear to inhibit cell-cell HIV-1 transmission [62]. In a more biologically relevant transwell system, SEs also blocked trans-infection from V428 cells to primary blood leukocytes (PBLs) [62].

The effects of SEs obtained from HIV-1-negative donors reduce HIV-1 infection by more than 50 % in a variety of *in vitro* cervical, monocytic and lymphocytic cell models, including PBLs [62, 63]. The levels of inhibition increase as the concentration of SE protein increases, plateauing at ~100 µg ml^−1^ [63]. The antiviral effect is conserved across HIV-1 viral strains (R5 and X4), including laboratory-adapted and transmitted founder isolates, and is effective when a range of viral inocula is studied [63]. Together, these data suggest that SEs inhibit HIV-1 in a donor-independent and dose-dependent manner, regardless of viral co-receptor tropism [63]. Most significantly, human SE-mediated reduction in HIV-1 infection is recapitulated *in vivo* using a murine-AIDS model of infection [62]. Infection with murine AIDS LP-BM5 virus at the murine vaginal mucosa showed that virus incubated with human SEs is reduced in viral replication and spread, as shown by reduced viral loads at the site of infection (vaginal epithelial cells) and in peripheral tissues (PBMCs, inguinal/subiliac draining lymph nodes and splenocytes), and that cell-free virus circulating in plasma is less infectious [62]. Murine vaginal epithelial cells internalized human SEs, and SE cargo (human APOBEC3G mRNA) was transferred to murine vaginal epithelial cells, suggesting that SEs may block infection at the vaginal mucosa [62].

### Semen exosomes inhibit HIV-1 infection

HIV-1 is notorious for its ability to circumvent control strategies and develop antiretroviral resistance. The high error rate of the HIV-1 polymerase allows the virus to select escape mutations in the presence of antiretroviral drugs that do not abrogate viral replication [136]. These mutations can occur at three main points during replication: (1) the conversion of viral ssRNA to dsDNA (reverse transcription), (2) the copying of integrated proviral DNA and (3) the transcription of viral RNA from proviral DNA [137]. HIV-1 also acquires adaptive mutations based on selection factors within different anatomical compartments. Viral populations in blood and the male genital tract differ in paired samples from chronically infected men [138], despite being identical during initial infection [138]. Therefore, combination control strategies that target different stages of the viral lifecycle have been highly successful at controlling the genetic diversity of HIV-1.

**Fig. 4.**
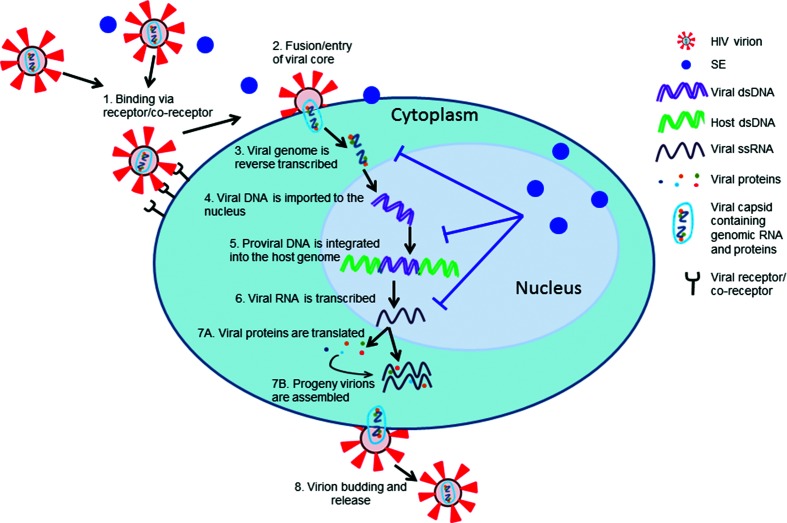
Semen exosomes inhibit HIV-1 lifecycle steps. Schematic of the HIV-1 lifecycle. (1) HIV-1 virions bind to cell receptor/co-receptor. (2) Fusion and entry inserts the viral core in the cell cytoplasm. (3) The viral genome is reverse transcribed from single-stranded RNA to double-stranded DNA. (4) Viral double-stranded DNA is imported into the cell nucleus. (5) Viral double-stranded DNA is integrated into host double-stranded DNA. (6) Viral RNA is transcribed from integrated DNA. Concurrently, (7A) viral proteins are translated from viral RNA and (7B) progeny virions are assembled with viral proteins and viral RNA. (8) New HIV-1 virions bud from the cell plasma membrane. Semen exosomes inhibit HIV-1 at the steps of reverse transcription, proviral integration and viral transcription. See text for references.

Although the exact mechanism(s) by which SEs inhibit HIV-1 infection is unknown, studies have shown that multiple steps in the HIV-1 lifecycle are affected by SEs, including: (1) the conversion of ssRNA to dsDNA (reverse transcription, RT), (2) the copying of integrated proviral DNA and (3) the transcription of viral RNA from proviral DNA [63, 139], as depicted in Fig. 4. Since three of these replication steps are key points where HIV-1 mutations arise, SEs may inhibit infection through multiple mechanisms [137]. SEs reduced HIV-1 proviral DNA and viral RNA levels in a variety of cell models across multiple HIV-1 strains [63]. These affects likely occured post-entry, as SEs did not reduce intracellular HIV-1 p24 capsid protein or RT activity levels 3 hours post-infection. In contrast, RT activity was reduced 24 hours post-infection [63]. RT is a potential lifecycle target of SEs, as SEs altered the ratio of RT subunits p51 and p66 by significantly reducing the levels of virion-associated p66 protein [63]. The ratio of RT subunits is important for the enzymatic activity of RT [63]. Murine AIDS virus infection implicates these same lifecycle steps. Specifically, the presence of human SEs reduced circulating murine AIDS virus LP-BM5 reverse transcriptase activity in cell-free blood plasma as well as viral DNA (proviral) and RNA levels in vaginal epithelial cells at the site of infection [62]. Despite there being no reduction in the levels of intracellular RT activity in cultured naïve murine splenocytes at 3 hours post-infection, SEs reduced RT activity by 24 hours post-infection, indicating that there is a post-entry inhibitory effect [62]. Whether SEs target each of these lifecycle steps independently or interfere with earlier viral lifecycle stages has yet to be determined.

It has been observed that SEs target viral factors and specifically block HIV-1 lifecycle events, even when viral binding and entry steps are bypassed [139]. SEs inhibit Tat-dependent transcription of a Tat expression vector and Tat exogenous protein. Specifically, SEs inhibit promoter activation of *de novo* Tat [139]. Thus SE-mediated anti-HIV-1 activity may directly target steps in the viral life cycle, in addition to influencing downstream replication events. SEs clearly inhibit the late steps of the viral lifecycle by reducing HIV-1 viral progeny RT, RNA and infectivity levels [62, 63]. In addition, the hypothesis that SE-mediated HIV-1 inhibition may involve multiple mechanisms is supported by mimicking different cell infection conditions during *in vivo* sexual transmission. Although virus entry was not inhibited when SEs were added to cells prior to HIV-1 infection, virus production was reduced in these cells regardless of whether SEs were added prior to, simultaneously with, or after the HIV-1 inoculum [63, 64]. SEs do not alter viral entry or release as determined by intracellular and progeny p24 protein content, respectively, suggesting that SEs mediate the inhibition of post-entry lifecycle steps [63]. Transcription-specific analyses show that SEs diminish HIV-1 promoter activity and reduce recruitment of transcription factors to the HIV-1 promoter, implicating a regulatory mechanism [139]. Taken together, these data suggest that SEs isolated from healthy donors inhibit HIV-1 at multiple stages of the viral lifecycle. It remains to be determined whether SEs isolated from the semen of HIV-infected individuals who are or are not on suppressive ART will control HIV infection of target cells.

It is known that HIV-1 acquires mutations to escape recognition by host immunity, yet mutations to the inhibitory effect of SEs have not been identified. Thus, HIV does not appear to be passively transmitted in semen in the male genital tract [138], and the anti-HIV effect of SEs may be conserved in SEs isolated from HIV-infected individuals, irrespective of ART. Recently, it was shown that antiretroviral drugs alter the cargo of exosomes isolated from HIV-1-infected cells cultured in the presence of drugs [140]. Similarly, chemotherapeutic drugs are packaged into exosomes, and such drugs were transferred to drug-naïve target cells [141–143]. It remains to be determined whether exosomes isolated from HIV-infected individuals on ART contain such drugs and if the drugs block HIV infection of target cells. Furthermore, it would be interesting to evaluate whether blood exosomes from HIV-infected individuals on ART display an ART-dependent anti-HIV phenotype that is not observed in blood exosomes from healthy donors [63]. Studies are underway to answer these and other pressing questions about the effect of HIV status on the antiviral phenotype of exosomes from body fluids.

### Semen exosome cargo

#### Semen exosome nucleic acid cargo

The heterogenous nature of SEs suggests that multiple cell types within the male reproductive tract contribute to the origin of these exosomes. This is supported by the finding that polyclonal antisera against SEs react with testes, epididymis, prostate and seminal vesicle accessory sex glands [144]. As such, SEs may contain a variety of coding and non-coding nucleic acids, proteins and lipid-associated cargoes [63, 133]. SE non-coding RNAs include microRNAs, Y RNAs and tRNAs that are postulated to act as regulatory signals within the mucosal microenvironment, and possibly contribute to the immune-regulatory profile of semen [133]. This may also contribute to immune cell trafficking during HIV-1 transmission, as SEs contain an abundance of miRNA that targets immune-related mRNAs [133]. The miRNA content is estimated to be one molecule per nine SE particles, thus SE appear to be relatively abundant. In contrast, there is an estimated one miRNA copy per 47 162 exosomes derived from blood plasma [145]. miRNA signatures among individual SE donors revealed 175 miRNAs present in all SE samples analysed. Let-7b, miR-148a and let-7a are the most abundant [133]. Previous studies demonstrated that let-7 miRNA family members target IL-10 mRNA, and that IL-10 inhibits cytotoxic T cell responses and contributes to T cell dysregulation during HIV-1 infection [146, 147]. Targeting by miR-148a can down-regulate MHC II expression, inhibit cytokine production and reduce T cell proliferation, further contributing to immune regulation during HIV-1 infection [148]. Therefore, the non-coding content of SEs may regulate immune functions influencing the proviral and antiviral milieu during infection. In addition to regulating the host immune response, SE nucleic acids may also modulate HIV-1 replication. In relation to the HIV-1 inhibitory phenotype of SE, the protein-coding RNA element of SEs may also be significant, as SEs contain HIV-1 restriction factors based on gene expression analyses [62, 63]. Although there appears to be some donor variability, SEs contain an abundance of APOBEC3 genes, including: A3C, A3D/E, A3F and A3G, as well as BST-2/tetherin [63]. APOBEC3 and BST-2/tetherin proteins are among the most extensively studied HIV-1 restriction factors [149, 150]. These restriction factors target viral products post-integration to control viral replication [149]. Importantly, SEs contain and transfer these factors to cells *in vivo* [62]. Analysis of murine vaginal cells exposed to human SEs showed exosome-mediated transfer of human A3G mRNA [62]. These observations suggest that SEs may regulate HIV-1 infection through the transfer of antiviral cargos.

### Semen exosome protein cargo

In addition to nucleic acid cargo, SEs contain an abundance of intra-vesicle and surface-associated proteins [64]. Analysis of the SE proteome identified >1000 proteins, including the exosome-associated markers ALIX and HSP70 [124]. Despite variation among donors, >300 common proteins among two groups of pooled donors were detected in SEs [124]. High physiological variation among individual donors in seminal composition may account for the relatively modest sharing of only 24 % of all proteins identified among SE donors. In addition, differences in isolation strategies and experimental processing may contribute to the number and species of proteins identified. Gene ontology (GO) analysis of the SE proteome revealed a link of SE proteins to biological processes. There is a correlation between SE-associated protein levels and SE-mediated function. For example, a correlation between HIV-1 infection and acetylcholine-esterase, CD9 and CD63 exosome surface protein levels is observed in different models of infection, and increased protein levels are correlated with decreased HIV-1 infectivity [64]. Although correlation does not prove causation, these studies suggest that SE-associated proteins may play a role in the anti-HIV activity of SEs. Determining the proteome of SE sub-populations may prove useful in narrowing down the cell types and tissues producing HIV inhibitory SEs within the male reproductive tract. Further, characterization of the source of the anti-HIV-1 cargo is important, as not all SE vesicles contain the same cargo [63, 64, 124]. In-depth comparative proteomics analyses of SEs with other non-inhibitory exosome proteomes, such as blood exosomes, may aid in identifying additional SE functions and antiviral mechanisms. This is not a general antiviral effect, as SEs did not inhibit herpes simplex virus-1 and −2 (HSV-1 and HSV-2, respectively) [63]. Despite the overlap between HIV-1 and HSV-1 and HSV-2 by virtue of being sexually transmitted viruses and their ability to persist quiescently in the host, HSV-1 and HSV-2 are DNA viruses with major differences in their life cycles compared to HIV-1. Although a flavivirus, the Zika virus (ZIKV) lifecycle is more comparable to HIV-1 as both are enveloped positive-sense single-stranded RNA viruses [151]. SEs inhibited ZIKV infection and attachment to target cells *in vitro*, a potential explanation for the low frequency of ZIKV sexual transmission despite the detection of high viral titres in semen [152].

### Semen exosome immunomodulatory functions

Although further understanding of the immunomodulatory capacity of SEs is needed, a few descriptions of the interaction of prostasomes with immune cells may provide some insight into SE-mediated immune regulation. Prostasomes, exosomes secreted from the prostate epithelium which are likely intermixed within SEs, bind to lymphocytes, inhibit lymphoproliferation and inhibit monocyte endocytosis *in vitro* [20]. This function is postulated to protect sperm from the hostile environment of the female reproductive tract during reproduction [153]. Prostasomes contain CD59, an inhibitor of the complement system, and are protected against complement-mediated cell lysis [154, 155]. Additionally, CD59 transferred from prostasomes to cells lacking CD59 *in vitro* maintain the ability to abrogate complement-mediated lysis [156]. In relation to viral infections, prostasomes are associated with CD46, a receptor for measles virus and a complement system co-factor. CD46 is a co-factor during the cleavage of C3b and C4b and regulates the complement cascade by inhibiting the formation of the membrane attack complex. CD46 is highly enriched in seminal plasma and is precipitated with prostasomes, suggesting a prostasome association [157]. During measles virus infection, CD46 is down-regulated from the cell surface, increasing the sensitivity of infected cells to complement-mediated lysis [158]. During reproduction, the complement inhibitors CD59 and CD46 may protect spermatozoa from complement attack in the female reproductive tract [157]. Although these complement inhibitors may also protect viruses from complement-mediated lysis, prostasomes have been described to inhibit measles virus activity [154]. Prostasomes and seminal plasma including prostasomes inhibit measles virus infectivity, possibly through viral binding to prostasome-associated CD46 [20, 154]. It is possible that this sub-population of SEs may impose an immunosuppressive effect during infection, although this has not yet been studied in the context of HIV-1 infection. Because prostasomes are speculated to temporarily reduce early immune responses, it is possible that SE-mediated immune regulation occurs early after HIV-1 infection [153].

Because the antiviral effect of SEs is cell-type independent, the effect of SEs during HIV-1 infection appears to interfere with the virus rather than the cell [63]. Therefore, it is likely that SEs may not alter HIV-1 target cell activation during infection. Studies are underway to determine the effect of SEs on lymphocyte activation and the associated cell proliferation and cytokine production. These studies will determine whether SEs induce viral reactivation in lymphocytes isolated from HIV-infected ART-suppressed individuals. Of note, SEs had no effect on viral reactivation in an *in vitro* cell model of HIV-1 latency [139].

### Semen exosomes and transcription factors

Exosomes in general are known to mediate proximal and distal cell signalling alterations, including changes in cellular transcription that permit disease progression [95, 159] or mitigation [139, 160]. The details on how exosomes achieve diverse roles in cells are unclear, but it is known that nucleic acid-binding proteins and transcription factor proteins are present in exosomes [161]. These proteins may therefore effect the exosome-directed phenotypic and functional changes observed. Indeed, it was recently shown that SEs block HIV-1 transcription initiation and elongation, and the recruitment of transcription factors [139]. Host transcription factors, including NF-kB, Sp1 and Pol II, as well as the viral factor Tat, are specific targets of SEs in an *in vitro* HIV infection model [139]. SEs reduce the DNA-binding ability of NF-kB, Sp1 and Pol II, resulting in reduced HIV-1 LTR activation by HIV-1 or by extracellular Tat. During infection, Tat associates with Sp1 and NF-kB, as well as other cellular factors, to drive HIV-1 LTR elongation [162, 163]. SEs specifically block the interaction of Tat with NF-kB p65 and Sp1 [139]. As a multifunctional protein, Tat regulates multiple steps of viral replication, including Pol II initiation of transcription, mRNA splicing and the functions of reverse transcriptase [164–170]. Tat is implicated as a probable target of SEs as SEs interfered in the DNA binding of Pol II, reduced the expression levels of HIV-1 mRNA splice variants and altered the ratio of HIV-1 RT subunits [63, 139]. Whether the effects of SEs on host and viral transcriptional regulators are distinct or related mechanisms is to be determined. In addition, these studies are not exhaustive, and other transcription factors may be affected. In support of the role of exosomes in transcriptional regulation, separate groups reported that a CD8 antiviral factor (CAF) secreted from CD8+ T cells restricts HIV replication at the level of viral transcription [171–173]. Although the identity of CAF is unknown, it has been suggested that exosomes contribute to these effects as exosomes released from CD8+ T cells suppressed HIV-1 [102].

## Discussion

Exosomes and enveloped viruses, including HIV-1, are highly similar in their physical and functional characteristics. Differentiation between the two types of vesicles during HIV-1 infection is difficult, but important for understanding their independent contributions to disease pathogenesis. Although the inhibitory or enhancing contribution of exosomes during HIV-1 infection seems to rely on the bio-fluid source, the inhibitory phenotype of exosomes from human semen may account for the low risk of infection per sexual exposure. The role of semen appears to vary considerably during infection, but the nucleic acid and proteinaceous content of semen exosomes may provide useful insights into the anti-HIV function of this particular subset of exosomes. It seems likely that semen exosomes target multiple steps of the HIV-1 lifecycle, and in addition may regulate the immune response to infection to restrict viral replication. Although recent data have allowed important progress to be made regarding our understanding of the interaction of these vehicles within the same biological system, many questions remain open, including identification of the antiviral factors contained within semen exosomes that inhibit HIV-1 and understanding of the mechanisms of SE perturbation of HIV-1 lifecycle events, as well as the immune-regulatory function of semen exosomes during infection. These questions are important for our understanding of the co-evolution of pathogenic (HIV-1) and protective (SE) vehicles of intercellular communication within the male reproductive tract.
